# Do Occupational and Patient Safety Culture in Hospitals Share Predictors in the Field of Psychosocial Working Conditions? Findings from a Cross-Sectional Study in German University Hospitals

**DOI:** 10.3390/ijerph15102131

**Published:** 2018-09-27

**Authors:** Anke Wagner, Antje Hammer, Tanja Manser, Peter Martus, Heidrun Sturm, Monika A. Rieger, on behalf of the WorkSafeMed Consortium

**Affiliations:** 1 Institute of Occupational and Social Medicine and Health Services Research, University Hospital of Tübingen, Wilhelmstraße 27, 72074 Tübingen, Germany; Anke.Wagner@med.uni-tuebingen.de (A.W.); Heidrun.Sturm@med.uni-tuebingen.de (H.S.); Monika.Rieger@med.uni-tuebingen.de (M.A.R.); 2 Institute for Patient Safety, University Hospital of Bonn, Sigmund-Freud-Str. 25, 53127 Bonn, Germany; Tanja.Manser@fhnw.ch; 3 FHNW School of Applied Psychology, University of Applied Sciences and Arts Northwestern Switzerland, Riggenbachstrasse 16, 4600 Olten, Switzerland; 4 Institute for Clinical Epidemiology and Applied Biometry, University Hospital of Tübingen, Silcherstraße 5, 72076 Tübingen, Germany; Peter.Martus@med.uni-tuebingen.de

**Keywords:** Germany, hospitals, occupational safety culture, patient safety culture, psychosocial working conditions, regression analysis, safety culture, transformational leadership

## Abstract

Background: In the healthcare sector, a comprehensive safety culture includes both patient care-related and occupational aspects. In recent years, healthcare studies have demonstrated diverse relationships between aspects of psychosocial working conditions, occupational, and patient safety culture. The aim of this study was to consider and test relevant predictors for staff’s perceptions of occupational and patient safety cultures in hospitals and whether there are shared predictors. From two German university hospitals, 381 physicians and 567 nurses completed a questionnaire on psychosocial working conditions, occupational, and patient safety culture. Two regression models with predictors for occupational and patient safety culture were conceptually developed and empirically tested. In the Occupational Safety Culture model, job satisfaction (β = 0.26, *p* ≤ 0.001), work‒privacy conflict (β = −0.19, *p* ≤ 0.001), and patient-related burnout (β = −0.20, *p* ≤ 0.001) were identified as central predictors. Important predictors in the Patient Safety Culture model were management support for patient safety (β = 0.24, *p* ≤ 0.001), supervisor support for patient safety (β = 0.18, *p* ≤ 0.001), and staffing (β = 0.21, *p* ≤ 0.001). The two models mainly resulted in different predictors. However, job satisfaction and leadership seem to play an important role in both models and can be used in the development of a comprehensive management of occupational and patient safety culture.

## 1. Introduction

In recent years, an increasing number of studies on safety culture have been carried out in the healthcare sector. Safety culture can be seen as part of the organizational culture and refers to “the product of individual and group values, attitudes, perceptions, competencies, and patterns of behaviour that determine the commitment to, and the style and proficiency of an organization’s health and safety management. Organizations with a positive safety culture are characterized by communications founded on mutual trust, by shared perceptions of the importance of safety and by confidence in the efficacy of preventive measures” [1].

The safety of healthcare workers and patients is a particular challenge, especially for hospitals. A comprehensive view of safety culture comprises both occupational and patient safety culture. Most studies, however, observed occupational and patient safety culture separately [for occupational safety culture see, e.g., [2,3] and for patient safety culture, e.g., [4,5,6,7,8,9]. Recently, several studies were conducted that included both constructs [10,11,12,13,14].

Previous studies on occupational and patient safety culture analysed associations between different aspects of working conditions, organizational culture, safety culture, patient and worker outcomes [10,11,12,13]. The research group of Hofman and Mark adapted a questionnaire for safety culture in industrial organizations and surveyed hospital nurses in the United States [10]. They found that safety culture predicted nurse back injuries, nurse satisfaction, and patient-related adverse events, like medication errors, urinary tract infections, and patient satisfaction [10]. A literature review found evidence that organizational climate influenced such nurse outcomes as, for example, less fluctuation among nurses, burnout, and job dissatisfaction [11]. There were also some tendencies showing that organizational climate was associated with patient outcomes, such as treatment errors and infections, but the authors stated that the results were inconsistent [11]. A survey of 723 American nurses by Taylor and colleagues showed that a poor safety culture was associated with injuries to both nurses and patients [12]. In another survey of 1866 clinical staff in Scotland, Agnew and colleagues again tested which dimensions of hospital safety climate were associated with patient and worker safety outcome measures (self-reported behaviour, patient injury, worker injuries), and also examined the influence of hospital climate perceptions on patient and worker-related safety outcomes [13]. In their study, Agnew and colleagues confirmed the previous results of Hofman et al. (2006) and Taylor et al. (2012).

A recently published study examined the relationship between occupational and patient safety culture [14]. Pousette and colleagues surveyed 1154 nurses, 886 assistant nurses, and 324 physicians in Sweden [14]. Their findings indicated that both kinds of safety culture had a strong positive correlation to each other. The authors concluded that integrated and coordinated interventions to improve safety culture should focus on occupational and patient safety together [14]. In an article published in 2005, Yassi and Hancock already proposed a comprehensive approach to safety culture that takes into account organizational factors and psychological and physical aspects of healthcare workers, among other things [15]. The authors stated that a comprehensive approach represents the best way to improve the healthcare workplace and as a consequence patient safety [15].

In summary, previous studies on occupational and patient safety culture in healthcare supported the presumed associations between working conditions and occupational and patient safety cultures. Yet, safety culture was often investigated with a special emphasis on either staff-related or patient-related injuries. In order to develop integrated interventions to manage occupational and patient safety culture, further research is needed on relevant predictors and their possible interrelationships. Currently, little or no research exists identifying predictors of both types of safety culture. However, identifying and examining similar and comprehensive predictors are important, because these predictors can be relevant components in a concept for the comprehensive integrated management of occupational and patient safety culture.

Therefore, we investigated the following research question in the current study: From the point of view of nurses and physicians, which predictors (from the areas of psychosocial working conditions, patient safety, and occupational safety) affect the occupational and patient safety culture in the hospital? The aim of our study was to consider and to identify potential predictors that were similar for both kinds of safety culture and whether there are shared predictors. After choosing relevant predictors for occupational and patient safety culture, models will be developed and tested simultaneously for the first time. Thus, we seek contributing to existing theories on occupational and patient safety and provide first insight in comprehensive and integrated models for occupational and patient safety culture to be tested in future studies. In long term, such models will help to identify common drivers for effective and resource efficient interventions in practice that serve both outcomes.

## 2. Materials and Methods

### 2.1. Study Design, Survey Instrument, and Data Collection

Between 2014 and 2017, we conducted the cross-sectional, bicentric, mixed-methods project “Working Conditions, Safety Culture and Patient Safety in Hospitals—What predicts the Safety of the Medication Process (WorkSafeMed)” [16]. Part of the WorkSafeMed project was a staff survey that formed the basis of the study presented here. The staff survey was conducted between April and July 2015, in two German university hospitals. Hospital selection was based on a convenient sample to have an appropriate sample size large enough to perform multivariate analyses and keep organizational characteristics as comparable as possible. We included inpatient units that treat at least 500 patients per year. We excluded intensive care and psychiatric units. In total, we collected data from 37 departments, including 73 units using a standardized paper-based questionnaire. The questionnaire was distributed to a total of 2512 physicians and nurses. We conducted at least one oral or written reminder one month after survey distribution.

The questionnaire included scales from common and validated instruments to measure psychosocial working conditions, transformational leadership, patient safety culture, and added self-constructed items to assess occupational safety culture:

- To measure psychosocial working conditions, we employed 17 scales of the German version of the Copenhagen Psychosocial Questionnaire (COPSOQ) [17,18]. We also adapted one scale from the Copenhagen Burnout Inventory (client-related burnout) [19] to measure patient-related burnout, and we used the short scale on Transformational Leadership (TLI-short) to assess transformational leadership [20,21].
- To capture different dimensions of patient safety culture, we focussed on the German version of the Hospital Survey on Patient Safety Culture (HSPSC-D) [22], and on a newly developed set of twin-items worded correspondingly to occupational safety culture items (TWINS Patient Safety).
- To measure different dimensions of occupational safety culture, we inserted three self-constructed indices (good Cronbach’s alpha from 0.76 to 0.82) and a set of twin items worded analogously to some patient safety culture items (TWINS Occupational Safety).

Prior to data collection, a pretest was carried out with 4 physicians and 8 nurses. An overview of all scales and items of the final questionnaire is provided in Table 1.

### 2.2. Ethics and Confidentiality Issues

Ethical approval was obtained from the ethical committees at the two participating university hospitals (reference numbers: #350/14 and #547/2014BO1). Informed consent was sought from participants, who were informed that the study was voluntary and that they could withdraw at any time. All data were analysed anonymously.

### 2.3. Statistical Analyses

Prior to data analyses, we imputed missing values in the survey data by grouping items into four separate imputation groups. Within each imputation group, respondents with missing values of >30% for scale items were excluded due to the limited data quality. Data for each imputation group were imputed with NORM 2.03 software using the Expectation-Maximization-algorithm [23,24].

In this study, statistical analyses included descriptive statistics, bivariate correlations, exploratory factor analysis, and stepwise multiple regression analysis [25]. Descriptive statistics were used to determine mean values and standard deviations of continuous variables and scale-scores, and absolute and percentage frequencies of categorical variables. Exploratory factor analysis was conducted to reduce and summarize the two twin-item sets for occupational and patient safety culture into four factors. In each case three items related to the direct supervisor formed the factors “occupational safety-related behaviour of the direct supervisor” (factor) and “patient safety-related behaviour of the direct supervisor” (factor). Also three items were used to create the following factors related to hospital management: “occupational safety-related behaviour of the hospital management” (factor) and “patient safety-related behaviour of the hospital management” (factor). In addition, two scales and two single items on patient safety culture (HSPSC-D) were combined into one factor that represents the “perceived patient safety” by physicians and nursing staff. The five factors we developed are shown in Table 2.

For further data analysis in the process, all scales and variables were orientated in the same direction to ensure a uniform interpretation of bivariate correlations and multiple regression analysis. Positive therefore means a favourable interpretation and negative is to be equated with an unfavourable interpretation. Prior to the stepwise multiple regression analysis, bivariate correlations (Pearson) were conducted to investigate the relationship between all content-relevant variables in the questionnaire (see Supplementary Materials, Table S1). Then, two regression models were developed on a conceptual basis, with regard to both, an Occupational Safety Culture model and a Patient Safety Culture model. All tests were two-sided and a *p*-value ≤ 0.05 was considered statistically significant. We checked the developed models for the following parameters: Durbin-Watson test, multicollinearity, and residuals for the evidence of bias [25]. Cluster effects were adjusted for by using Generalized Estimating Equations (GEE) [26]. The specific type was GEE 1 with IEE (Independence Estimating Equations). This method is a robust approach applicable if cluster effects are nuisance parameters and not of scientific interest, which is the case in our study. Essentially GEE 1 with IEE leaves regression parameter estimates unchanged but corrects the standard errors for cluster effects. In multiple regression models with variable selection the set of chosen variables might change compared to the naïve analysis without correction after application of GEE. Data were analysed using IBM Statistics SPSS for Windows, version 25 (IBM Corp., Armonk, NY, USA).

To develop the Occupational Safety Culture model, different scales wer e taken from the questionnaire since we assumed that well-designed psychosocial working conditions and leadership impact on perceived occupational safety culture. Therefore, we used the following scales, factors and single items from the areas of general psychosocial working conditions (stress, according strain, leadership), and occupational safety dimensions as independent variables (predictors):

- Stress: “quantitative demands” (COPSOQ), “emotional demands” (COPSOQ), “work-privacy-conflict” (COPSOQ),
- Strain: “job satisfaction” (COPSOQ) and “patient-related burnout” (adapted from CBI),
- Leadership focusing on a specific leadership style: “transformational leadership” (TLI-short),
- Leadership with regard to occupational safety: “supervisor support for occupational safety” (TWINS Occupational Safety), “occupational safety-related behaviour of the direct supervisor” (factor—TWINS Occupational Safety), “occupational safety-related behaviour of the hospital management” (factor—TWINS Occupational Safety), and
- Occupational safety dimension: “individual influence on how well occupational safety is implemented at the workplace” (single item—TWINS Occupational Safety).

The self-developed index “personal perception of the frequency of occupational risks” (see Table 1) represented the dependent variable, as we considered it a good indicator of perceived occupational safety culture.

The Patient Safety Culture model was developed analogously to the Occupational Safety Culture model. As for the Occupational Safety Culture model we presumed that well-designed psychosocial working conditions and leadership impact on perceived patient safety culture. However, the rather unspecific COPSOQ scales were not integrated into the Patient Safety Culture model, with the exception of the scale job satisfaction. Instead, HSPSC-D scales with similar contents as the respective COPSOQ scales but with a specific reference to patient safety were included in the model. We also used the following scales as independent variables (predictors) from the areas of psychosocial working conditions (strain, leadership), and patient safety dimensions:

- Strain: “job satisfaction” (COPSOQ), “patient-related burnout” (adapted from CBI),
- Leadership focusing on a specific leadership style: “transformational leadership” (TLI short),
- Leadership with regard to patient safety: “management support for patient safety” (HSPSC-D), “supervisor support for patient safety” (TWINS Patient Safety), “patient safety-related behaviour of the direct supervisor” (factor—TWINS Patient Safety), “patient safety-related behaviour of the hospital management” (factor—TWINS Patient Safety),
- Patient safety dimensions: “staffing” (HSPSC-D), “feedback and communication about error” (HSPSC-D), “organizational learning” (HSPSC-D), “handoffs and transitions” (HSPSC-D), and “individual influence on how well patient safety is implemented at the workplace” (single item—TWINS Patient Safety)

The factor “perceived patient safety” (see Table 2) represented the dependent variable.

## 3. Results

### 3.1. Descriptive Results

Out of 2512 distributed questionnaires, 995 (39.6%) were completed and returned. The characteristics of the sample are summarized in Table 3. In particular, the following socio-demographic characteristics were requested: profession, gender, age, supervisor function, and professional experience in years. Overall, there were more nurses than doctors in our sample. The mean age of the participants was 37.67 years (SD = 10.69), and the average professional experience was 13.49 years (SD = 10.91) (see Table 3).

### 3.2. Occupational Safety Culture Model

#### 3.2.1. The Association between Psychosocial Working Conditions, Occupational Safety Dimensions, and Occupational Safety Culture

As shown in Table 4, there were significant associations between all independent variables and the dependent variable. Significant negative associations between our indicator for perceived occupational safety culture and independent variables were found for “quantitative demands” (r = −0.25, *p* = 0.000), “emotional demands” (r = −0.23, *p* = 0.000), “work‒privacy conflict” (r = −0.33, *p* = 0.000), and “patient-related burnout” (r = −0.35, *p* = 0.000). We can therefore state that increasing values for these parameters were accompanied by a lower rating of the perceived occupational safety culture. Significant positive associations were found for “job satisfaction” (r = 0.40, *p* = 0.000), “transformational leadership” (r = 0.21, *p* = 0.000), “supervisor support for occupational safety” (r = 0.23, *p* = 0.000), “occupational safety-related behaviour of the direct supervisor” (r = 0.20, *p* = 0.000), “occupational safety-related behaviour of the hospital management” (r = 0.24, *p* = 0.000), and the “individual influence on how well occupational safety is implemented at the workplace” (r = 0.21, *p* = 0.000).

#### 3.2.2. The Independent Variables Influencing Occupational Safety Culture

A stepwise multiple regression analysis was then carried out to identify relevant predictors for the perceived occupational safety culture (see Table 5). The following significant predictors prevailed: “job satisfaction” (β = 0.26, *p* ≤ 0.001), “patient-related burnout” (β = −0.20, *p* ≤ 0.001), and “work-privacy-conflict” (β = −0.19, *p* ≤ 0.001), and “individual influence on how well occupational safety is implemented at the workplace” (β = 0.08, *p* ≤ 0.01). Overall, the Occupational Safety Culture model achieved an explained variance of 0.27 *R*^2^.

### 3.3. Patient Safety Culture Model

#### 3.3.1. The Association between Psychosocial Working Conditions, Patient Safety Dimensions, and Patient Safety Culture

As shown in Table 6, we found one significant negative association between “perceived patient safety” and “patient-related burnout” (r = −0.30, *p* = 0.000). Only significant positive associations were found for all other variables. The highest positive correlations were found for the following variables: “management support for patient safety” (r = 0.66, *p* = 0.000), “organizational learning” (r = 0.60, *p* = 0.000), “supervisor support for patient safety” (r = 0.57, *p* = 0.000), “feedback and communication about error” (r = 0.55, *p* = 0.000), “patient safety-related behaviour of the hospital management” (r = 0.55, *p* = 0.000), “staffing” (r = 0.52, *p* = 0.000), and “job satisfaction” (r = 0.54, *p* = 0.000).

#### 3.3.2. The Independent Variables Influencing Patient Safety Culture

The stepwise multiple regression analysis revealed the following significant predictors for the dependent variable “perceived patient safety”: “management support for patient safety” (β = 0.24, *p* ≤ 0.001), “staffing” (β = 0.21, *p* ≤ 0.001), “supervisor support for patient safety” (β = 0.18, *p* ≤ 0.001), “organizational learning” (β = 0.14, *p* ≤ 0.001), “feedback and communication about error” (β = 0.14, *p* ≤ 0.001), “individual influence on how well patient safety is implemented at the workplace” (β = 0.13, *p* ≤ 0.001), “handoffs and transitions” (β = 0.12, *p* ≤ 0.001), “patient safety-related behaviour of the direct supervisor” (β = −0.08, *p* ≤ 0.01), and “job satisfaction” (β = 0.06, *p* ≤ 0.05) (see Table 7). Particularly relevant predictors were “management support for patient safety,” “supervisor support for patient safety,” and “staffing”. Overall, the Patient Safety Culture model achieved an explained variance of 0.64 *R*^2^.

### 3.4. Comparison of the Shared Predictors Used in the Occupational Safety Culture model and in the Patient Safety Culture Model

In another comparison using the previous bivariate correlation analysis, the following independent variables were contrasted: “job satisfaction” (COPSOQ), “patient-related burnout” (adapted from CBI), and “transformational leadership” (TLI short). We also reviewed and compared the bivariate correlations to the TWINS on Occupational and Patient Safety Culture. The comparison demonstrated that the correlations of the independent variables to our dependent variable for Patient Safety Culture performed better than the correlations of the independent variables to the dependent variable for Occupational Safety Culture (see Table 8).

An additional correlation analysis (Pearson) performed between the dependent variable for Occupational Safety Culture (“personal perception of the frequency of occupational risks”) and the dependent variable for Patient Safety Culture (“perceived patient safety”) revealed a significant positive association (r = 0.352, *p* = 0.000). The correlation coefficient corresponded to a medium effect.

## 4. Discussion

In this study, we investigated potential predictors as similar as possible in the field of psychosocial working conditions, occupational and patient safety culture. Based on the selected predictors, models for occupational and patient safety culture were developed and tested simultaneously for the first time. Identifying and examining similar and comprehensive predictors is important, because these predictors can be used in the ongoing discussion for developing a comprehensive and integrated management of occupational and patient safety culture. Our results allow first insight for identifying common drivers—e.g., the concept of common education and training addressing both, occupational and patient safety culture in health care—to support effective and resource efficient interventions that serve both outcomes.

### 4.1. Occupational Safety Culture Model

In the correlation analyses, we discovered negative associations between demands, work-privacy-conflict, patient-related burnout, and our dependent variable to depict perceived occupational safety culture (“Personal perception of the frequency of occupational risks”). Based on these finding, we can conclude that more stressful psychosocial working conditions, such as high demands and increased risk for burnout, go hand in hand with a lower perception of occupational safety culture. Unfortunately, there are few studies in the healthcare sector that have investigated these links between demands, work‒privacy conflict, patient-related burnout, and occupational safety culture. A previous study with 250 nurses investigated the relationship between occupational burnout and safety climate in the workplace [3]. They found a significant negative correlation between safety climate and all dimensions of occupational burnout. As a consequence, a higher occupational burnout implied a lower level of safety climate, and nurses with no or lower stress had a better perception of safety climate [3].

In our correlation analyses, positive associations were found for “job satisfaction”, “transformational leadership”, “supervisor support for occupational safety”, “occupational safety-related behaviour of the direct supervisor”, “occupational safety-related behaviour of the hospital management”, and the “individual influence on how well occupational safety is implemented at the workplace”. Thus, job satisfaction as a variable for strain and positively experienced leadership style and behaviour seem to contribute to a favourable occupational safety culture. Zarei and colleagues also found that nurses with higher job satisfaction and higher job interest had a better perception of safety climate in the workplace [3]. Another study investigated the link between leadership and safety outcomes in hospitals and conducted a survey with 600 nurses [27]. This study showed the positive association of resonant leadership and interactional justice on relationships, quality of work environment, and specific outcomes of safety climate, e.g., decreased reported medication errors, intentions to leave, and emotional exhaustion [27].

In our tested Occupational Safety Culture model, “job satisfaction,” “work‒privacy conflict,” and “patient-related burnout” were identified as central predictors of “perceived occupational safety culture.” Surprisingly, transformational leadership and the other scales of leadership with regard to occupational safety are not relevant in the model. A possible explanation for this result might be that the variable “job satisfaction” in the single items partly includes some attitudes to leadership as an indirect question. This could be the reason why other scales for leadership do not play a role in the model. Based on the identified predictors in the model, the promotion of high job satisfaction and the reduction of psychosocial strain and stress such as patient-related burnout and work‒privacy conflict could contribute to an improved occupational safety culture. Hospital work is currently characterized by high demands in the field of psychosocial working conditions, especially in Germany. There is an increasing number of patients with multimorbidity and need for care. At the same time, there is a high shortage of nurses and physicians in the health care system. This could lead to high demands and stress for nurses and physicians. In our opinion, this development can also impede or even hinder the implementation and improvement of an occupational safety culture.

In summary, our investigated predictors in the Occupational Safety Culture model explained only 27% of the variance. Due to this rather low model quality, we assume that the occupational safety culture was insufficiently captured in our questionnaire and that essential predictors are still missing in the model. Flin already proposed a model with important elements for occupational safety culture in healthcare [28]. Elements in the model were derived from research in industrial settings and based on organisational aspects (e.g., perceptions of management and supervisor, prioritisation of safety), motivational aspects (e.g., expectations regarding outcomes for particular behaviours), unsafe behaviours (e.g., not taking precautions, rule breaking, risk taking, not speaking up, not reporting incidents/near misses), and errors (e.g., worker injury) [28]. Our questionnaire focussed mainly on organisational aspects with the perceptions of management and supervisors regarding safety issues. So, in future studies, our questionnaire should be augmented to question and analyse more motivational aspects, unsafe behaviours, and specific worker injuries in the hospital setting.

### 4.2. Patient Safety Culture Model

In our correlation analysis, we found one significant negative association between “perceived patient safety” and “patient-related burnout.” Similar to the Occupational Safety Culture model, patient-related burnout as indicator for psychosocial strain also seemed to be accompanied by a lower perception of patient safety culture. However, our study only covered one dimension of burnout. Therefore, this result can only be compared with other studies to a very limited extent. The negative relationship between burnout and a lower perception of patient safety culture has been confirmed in other studies e.g., [29,30,31,32,33]. In 2008, Halbesleben and colleagues questioned 148 nurses and showed an association between burnout and the perception of a lower patient safety culture and an unsafe environment [29]. Alves and colleagues conducted a correlation study with 267 nurses and found that a lower level of emotional exhaustion was accompanied by a more positive perception of the patient safety climate [30,31]. Profit et al. investigated the relationship between burnout and patient safety culture in neonatal intensive care units [32]. They questioned 2073 nurses, nurse practitioners, respiratory care providers, and physicians. As a result, neonatal intensive care units with more burnout had a lower teamwork climate and a lower patient safety climate [32]. Vifladt and colleagues also conducted a study in intensive care units with 143 nurses and confirmed the previous results. A favourable safety culture was therefore associated with the absence of burnout [33].

Only significant positive associations were found for all other variables in the correlation analyses. Positive correlations were found for “management support for patient safety,” “supervisor support for patient safety,” and “patient safety-related behaviour of the hospital management.” This shows the importance of leadership for a patient safety culture. Positive correlations were also evident in “organizational learning,” “feedback and communication about error,” “staffing”, and “job satisfaction.” Alves and colleagues were also able to confirm the positive correlation between better work environment, higher job satisfaction and a more positive judgment of the patient safety climate [30,31].

In the tested Patient Safety Culture model the following predictors prevailed:

- predictors about work-related psychosocial strain (job satisfaction),
- predictors about leadership with regard to patient safety, e.g., management support for patient safety (scale), supervisor support for patient safety (scale), patient safety-related behaviour of the direct supervisor (factor),
- predictors about patient safety dimensions, e.g., staffing (scale), feedback and communication about error (scale), organizational learning (scale), handoffs and transitions (scale), and individual influence on how well patient safety is implemented at the workplace (single item).

Studies to date confirm these results in part, and reveal the importance of certain scales for patient safety culture. For example Alves and colleagues found that the job satisfaction variable was predictive of safety climate [31]. In our study, the impact of the scale job satisfaction on our dependent variable, perceived patient safety, was very low. This may be because the job satisfaction scale from the COPSOQ questionnaire is already better represented in other HSPSC scales with a specific emphasis on patient safety (e.g., support for patient safety, staffing, organizational learning and, feedback and communication about error).

A recently conducted review examined the relationship between nurse working conditions and patient outcomes, and reported the association between staffing, resource adequacy, and patient outcomes [34]. Aiken and colleagues conducted the well-known RN4CAST study. As part of their study, they demonstrated the relationship between an increase in workload and the likelihood of hospital mortality in different European countries [35]. The results imply that nurse staffing cuts might adversely affect patient outcomes [35]. Schubert and colleagues explored the relationship between rationing of nursing care and inpatient mortality in Swiss hospitals [36]. The results of the study revealed that patients treated in hospitals with an increased rationing level and a high patient-to-nurse ratio had a higher risk of mortality [36].

To improve the perceived patient safety culture for nurses and physicians, the following measures can be derived from the tested predictors. Managers and direct supervisors play a pivotal role and should be supported in implementing a patient safety culture. In addition, dealing with errors, open communication, and feedback can contribute to improving patient safety culture. The results imply that adequate staffing and other factors, such as organisational learning, have a high influence on how patient safety culture is experienced by both nurses and physicians. The chosen predictors in the model explained 64% of the variance. In general, the Patient Safety Culture model demonstrated a high and satisfying model quality.

### 4.3. Summary and Implications for a Comprehensive Integrated Management of Occupational and Patient Safety Culture

We identified and tested different potential predictors in the area of psychosocial working conditions, occupational, and patient safety with an impact on perceived occupational and patient safety culture. The initial correlation analyses revealed that job satisfaction and leadership were associated with higher and patient-related burnout with a lower occupational and patient safety culture. The Patient Safety Culture model showed a high and satisfactory quality in contrast to the Occupational Safety Culture model. General job satisfaction was the only significant predictor in both tested models, but the impact in the Patient Safety Culture model was comparably very low. Leadership (support and role model function) was also identified as an important indirect (occupational safety culture—modifying job satisfaction) or direct (patient safety culture) predictor in both models. In summary, job satisfaction and leadership seem to play a crucial role and should therefore be considered in a comprehensive integrated management of both, occupational and patient safety culture. However, further studies are needed to confirm our results.

### 4.4. Strengths and Limitations

For the first time, an attempt was made to develop models for occupational and patient safety culture simultaneously and to present interrelationships. The two models were developed in such a way that each model is as broad as possible in content but still empirically verifiable. By looking at two kinds of safety culture in parallel, a deeper understanding of overarching and specifically relevant predictors can be achieved. For professional practice, this means that communication on these topics and intervention approaches can benefit more specifically from synergies and specific influencing factors can also be taken into account.

There were also some limitations in our study. First, the survey was conducted at one point in time at two university hospitals in Germany. So—despite the rather good response rate of 39.6%—the results were neither representative for other university hospitals nor all hospitals in Germany. Thus, the generalizability for other healthcare contexts (type of hospital, healthcare sector, country) is unknown and need to be considered in further studies. In addition, the results were based on self-reports with highly subjective judgements in the survey data.

Second, we pursued a highly theoretical and explorative approach with a cross sectional design while developing the two models, so we cannot discuss causality. Even so, for drawing causal conclusions, further research is required to study the nature of these relationships using longitudinal studies. Moreover, we chose stepwise regression analyses to test our models. Even, this method is acceptable for exploratory model building [25], in future research different types of variable inclusion should be considered for further developments of these models [37]. Nonetheless, our data allow a first insight in comprehensive models for occupational and patient safety culture and point towards common predictors, providing an important base for future research.

Third, the two professional groups were analysed together in the regression analysis. In the future, it may be worthwhile to develop models separately according to each professional group. These models could be used to derive occupational group-specific interventions and improvements.

Fourth, variables of the two models were measured with different constructs, as no differentiated measure exists for occupational and patient safety so far. While patient safety culture was measured with the specific and established HSPSC-D, which also assesses the perception of some working conditions (e.g., staffing) in relation to patient safety, no such specific measure in relation to occupational safety was available. Instead, psychosocial working conditions were measured with the more generic COPSOQ-questionnaire. Hence, the analyses of the possible impact of working conditions with regard to occupational and patient safety culture resulted in different models: in the Patient Safety Culture model, several constructs from the more generic measure were excluded, but from variables from the more specific measure were retained. However, the results show, that basic assumptions (e.g., for Transformational leadership and Job satisfaction) remain relevant. In future research the development of more differentiated measures on working conditions with regard to occupational and patient safety should be considered for improving our models. Moreover, as we focussed solely on transformational leadership, other leadership styles, such as transactional leadership, relationship-based approaches or laissez faire might be worth to consider.

Finally, another limitation lies in the Occupational Safety Culture model. Essential predictors seemed to be missing in this model; we achieved a rather low model quality. The additional comparison of some independent variables used in both models revealed for example that the correlation analyses for the dependent variable for patient safety (“perceived patient safety”) performed better than the correlation analyses for the dependent variable for occupational safety (“personal perception of the frequency of occupational risks”). We also found only a medium‒strong association between the dependent variables for the two models. In principle, there are two possible interpretations of our findings: (1) Both concepts, occupational and patient safety culture are only moderately related to each other and thus do not share common predictors. However, due to the overall limited predictability of occupational safety culture we favor a different interpretation: (2) The operationalization of occupational safety culture has not been successful, and by improving the measurement of this concept, we would find stronger correlations of the two concepts and a larger number of shared predictors.

## 5. Conclusions

In our study we found a good predictability of patient safety, but not of occupational safety using established and novel predictors. Moreover, we only found a limited number of shared predictors of both concepts. However, the identified predictors (job satisfaction and leadership) might be useful for the ongoing discussion and later development of a concept for the comprehensive integrated management of occupational and patient safety culture. Additionally, we hypothesize that operationalization of occupational safety has to be improved. Further studies should focus not only on safety culture, but also on outcomes relevant to patients and staff, like specific indicators for safety [38]. These studies should also analyse which predictors are relevant for perceived occupational safety culture in the hospital setting. Answering these questions can support the integrated management of occupational and patient safety culture, and the further holistic development of a safety culture in the hospital setting.

## Figures and Tables

**Table 1:** Overview of scales and items of the questionnaire.

Topic	Instrument	Scales/Indices/Single Items	Interpretation
Psychosocial working conditions	COPSOQ ^1^ [17,18]	Quantitative demands (scale, 4 items)	High = negative
		Emotional demands (scale, 3 items)	High = negative
		Work-privacy-conflict (scale, 5 items)	High = negative
		Influence at work (scale, 4 items)	High = positive
		Degree of freedom at work (scale, 4 items)	High = positive
		Possibilities for development (scale, 4 items)	High = positive
		Meaning of work (scale, 3 items)	High = positive
		Workplace commitment (scale, 4 items)	High = positive
		Predictability (scale, 2 items)	High = positive
		Role clarity (scale, 4 items)	High = positive
		Role conflicts (scale, 4 items)	High = negative
		Social relations (scale, 2 items)	High = positive
		Feedback (scale, 2 items)	High = positive
		Social support (scale, 4 items)	High = positive
		Sense of community (scale, 3 items)	High = positive
		Quality of leadership (scale, 4 items)	High = positive
		Job satisfaction (scale, 7 items)	High = positive
	adapted from CBI ^2^ [19]	Patient-related burnout (scale, 6 items)	High = negative
	TLI short ^3^ [20,21]	Transformational leadership (scale, 6 items)	High = positive
Patient safety dimensions	HSPSC-D ^4^ [22]	Teamwork within units (scale, 4 items)	High = positive
		Staffing (scale, 4 items)	High = positive
		Organizational learning (scale, 3 items)	High = positive
		Nonpunitive response to error (scale, 3 items)	High = positive
		Supervisor/ manager expectations (scale, 4 items)	High = positive
		Feedback and communication about error (scale, 3 items)	High = positive
		Communication openness (scale, 3 items)	High = positive
		Management support for patient safety (scale, 3 items)	High = positive
		Teamwork across units (scale, 4 items)	High = positive
		Handoffs and transitions (scale, 4 items)	High = positive
		Frequency of event reported (scale, 3 items)	High = positive
		Overall perceptions of patient safety (scale, 4 items)	High = positive
		Patient safety grade (single item)	Low = positive
		Safety grade in the medication process (single item)	Low = positive
	TWINS Patient Safety ^4^	Supervisor support for patient safety (scale, 3 items)	High = positive
		My direct supervisor openly addresses problems concerning patient safety in our hospital (single item)	High = positive
		My direct supervisor focuses more on patient safety than a year ago (single item)	High = positive
		It is important to my direct supervisor that our hospital pays great attention to patient safety (single item)	High = positive
		Hospital management openly addresses problems concerning patient safety in our hospital (single item)	High = positive
		Hospital management focuses more on patient safety than a year ago (single item)	High = positive
		It is important to the hospital management that our hospital pays great attention to patient safety (single item)	High = positive
		Do you have an individual influence on how well patient safety is implemented at the workplace? (single item)	Low = positive
Occupational safety dimensions	Self-developed indices ^5^	Subjective assessment of specific protective measures (behaviour and regulations) related to infectious diseases (index, 7 items)	Low = positive
		Subjective assessment of occupational safety measures initiated by the employer, related to own safety (index, 6 items)	Low = positive
		Personal perception of the frequency of occupational risks (index, 4 items)	High = positive
	TWINS Occupational Safety ^5^	Supervisor support for occupational safety (scale, 3 items)	High = positive
		My direct supervisor openly addresses problems concerning occupational safety in our hospital (single item)	High = positive
		My direct supervisor focuses more on occupational safety than a year ago (single item)	High = positive
		It is important to my direct supervisor that our hospital pays great attention to occupational safety (single item)	High = positive
		Hospital management openly addresses problems concerning occupational safety in our hospital (single item)	High = positive
		Hospital management focuses more on occupational safety than a year ago (single item)	High = positive
		It is important to the hospital management that our hospital pays great attention to occupational safety (single item)	High = positive
		Do you have an individual influence on how well occupational safety is implemented at the workplace? (single item)	Low = positive

^1^ COPSOQ scales (possible range 1–4 or 1–5), before calculating scale scores, scales were transformed into scores ranging from 0 (minimum value, “do not agree at all”) to 100 points (maximum value, “fully agree”). ^2^ CBI scale (possible range 1–5), before calculating scale scores, scales were transformed into scores ranging from 0 (minimum value, “do not agree at all”) to 100 points (maximum value, “fully agree”). ^3^ TLI short scale (possible range 1–5). ^4^ HSPSC-D scales, TWINS Patient Safety single items (possible range 1–5). ^5^ Self-developed indices, TWINS Occupational Safety single items (possible range 1–5).

**Table 2:** Results of the exploratory factor analysis.

Source	Single Items	Constructed Factor
TWINS Occupational Safety	- My direct supervisor openly addresses problems concerning occupational safety in our hospital (single item) - My direct supervisor focuses more on occupational safety than a year ago (single item) - It is important to my direct supervisor that our hospital pays great attention to occupational safety (single item)	Factor “Occupational safety-related behaviour of the direct supervisor”
TWINS Occupational Safety	- Hospital management openly addresses problems concerning occupational safety in our hospital (single item) - Hospital management focuses more on occupational safety than a year ago (single item) - It is important to the hospital management that our hospital pays great attention to occupational safety (single item)	Factor “Occupational safety-related behaviour of the hospital management”
TWINS Patient Safety	- My direct supervisor openly addresses problems concerning patient safety in our hospital (single item) - My direct supervisor focuses more on patient safety than a year ago (single item) - It is important to my direct supervisor that our hospital pays great attention to patient safety (single item)	Factor “Patient safety-related behaviour of the direct supervisor”
TWINS Patient Safety	- Hospital management openly addresses problems concerning patient safety in our hospital (single item) - Hospital management focuses more on patient safety than a year ago (single item) - It is important to the hospital management that our hospital pays great attention to patient safety (single item)	Factor “Patient safety-related behaviour of the hospital management”
HSPSC-D [22]	- Frequency of event reported (scale, three items) - Overall perceptions of patient safety (scale, four items) - Patient safety grade (single item) - Safety grade in the medication process (single item)	Factor “Perceived patient safety”

**Table 3:** Demographic characteristics of study respondents.

Characteristic	Responders	
	*n*	% ^1^
**Profession**		
Nurse	567	57.0%
Physician	381	38.3%
Others	19	1.9%
Missing	28	2.8%
**Gender**		
Male	291	29.2%
Female	656	65.9%
Missing	48	4.8%
**Age, mean (SD) years**	37.67 (10.69)	
Missing	90	
**Supervisor function**		
Yes	195	19.6%
No	759	76.3%
Missing	41	4.1%
**Professional experience, mean (SD) years**	13.49 (10.91)	
Missing	61	

^1^ Percentages do not sum up to 100% due to rounding.

**Table 4:** Occupational Safety Culture model—correlations of independent variables and the outcome “personal perception of the frequency of occupational risks”.

Independent Variables (Scales and Factors)	Dependent Variable ^1^ (Index)		
	Pearson correlation	Sig0. (2-tailed)	N
***COPSOQ—Psychosocial working conditions***			
Quantitative demands (scale)	−0.25 **	0.000	970
Emotional demands (scale)	−0.23 **	0.000	970
Work-privacy-conflict (scale)	−0.33 **	0.000	970
Job satisfaction (scale)	0.40 **	0.000	970
***CBI—Patient-related burnout***			
Patient-related burnout (scale)	−0.35 **	0.000	970
***TLI short—Transformational leadership***			
Transformational leadership (scale)	0.21 **	0.000	940
***TWINS Occupational Safety—Occupational Safety Culture***			
Supervisor support for occupational safety (scale)	0.23 **	0.000	940
Occupational safety-related behaviour of the direct supervisor (factor)	0.20 **	0.000	940
Occupational safety-related behaviour of the hospital management (factor)	0.24 **	0.000	940
Individual influence on how well occupational safety is implemented at the workplace (single item)	0.21 **	0.000	940

^1^ Dependent variable: personal perception of the frequency of occupational risks (index). ** Correlation is significant at the 0.01 level (two-tailed).

**Table 5:** Occupational Safety Culture model—stepwise linear regression analysis adjusted for cluster effects.

Variable Group	Variables	B	SE	β	Chi−Square	*p*
	Constant	3.01	0.21		197.056	0.000
***Psychosocial working conditions***	Job satisfaction (scale)	0.02	0.00	0.26	54.981	0.000
	Work−privacy conflict (scale)	−0.01	0.00	−0.19	33.513	0.000
	Patient-related burnout (scale)	−0.01	0.00	−0.20	37.331	0.000
***Occupational Safety Culture***	Individual influence on how well occupational safety is implemented at the workplace (single item)	0.06	0.02	0.08	7.830	0.005

*N* = 921, *R^2^* = 0.27, Adj. *R^2^* = 0.27. Dependent variable: personal perception of the frequency of occupational risks (index); Adjustment for cluster effects via Generalized Estimating Equations (GEE).

**Table 6:** Patient Safety Culture model—Correlations of independent variables and the outcome “perceived patient safety”.

Independent Variables (Scales and Factors)	^1^ Dependent Variable (Factor)		
	Pearson correlation	Sig. (2-tailed)	N
***COPSOQ—Psychosocial working conditions***			
Job satisfaction (scale)	0.54 **	0.000	971
***CBI—Patient-related burnout***			
Patient-related burnout (scale)	−0.30 **	0.000	971
***TLI short—Transformational leadership***			
Transformational leadership (scale)	0.39 **	0.000	949
***HSPSC-D and TWINS Patient Safety—Patient Safety Culture***			
Staffing (scale)	0.52 **	0.000	974
Management support for patient safety (scale)	0.66 **	0.000	974
Organizational learning (scale)	0.60 **	0.000	974
Feedback and communication about error (scale)	0.55 **	0.000	974
Handoffs and transitions (scale)	0.44 **	0.000	974
Supervisor support for patient safety (scale)	0.57 **	0.000	949
Patient safety-related behaviour of the direct supervisor (factor)	0.45 **	0.000	949
Patient safety-related behaviour of the hospital management (factor)	0.55 **	0.000	949
Individual influence on how well patient safety is implemented at the workplace (single item)	0.46 **	0.000	949

^1^ Dependent variable (factor): perceived patient safety (factor). ** Correlation is significant at the 0.01 level (two-tailed).

**Table 7:** Patient Safety Culture model—stepwise linear regression analysis adjusted for cluster effects.

Variable Group	Variables	B	SE	β	Chi-Square	*p*
	Constant	−4.03	0.24		275.8	0.000
***Psychosocial working conditions***	Job satisfaction (scale)	0.01	0.002	0.06	4.94	0.026
***Patient Safety Culture***	Management support for patient safety (scale)	0.28	0.03	0.24	72.1	0.000
	Supervisor support for patient safety (scale)	0.24	0.05	0.18	27.5	0.000
	Staffing (scale)	0.27	0.03	0.21	95.7	0.000
	Organizational learning (scale)	0.21	0.04	0.14	23.0	0.000
	Feedback and communication about error (scale)	0.16	0.03	0.14	22.4	0.000
	Individual influence on how well patient safety is implemented at the workplace (single item)	0.13	0.02	0.13	29.8	0.000
	Handoffs and transitions (scale)	0.18	0.04	0.12	19.2	0.000
	Patient safety-related behaviour of the direct supervisor (factor)	−0.08	0.03	−0.08	6.64	0.010

*N* = 945, *R*^2^ = 0.65, Adj. *R*^2^ = 0.64. Dependent variable (factor): perceived patient safety (factor), Adjustment for cluster effects via Generalized Estimating Equations (GEE).

**Table 8:** Correlations of independent variables used in both models.

	Associations between Independent Variables and the Dependent Variable ^1^ for Occupational Safety Culture			Associations between Independent Variables and the Dependent Variable ^2^ for Patient Safety Culture		
	Pearson Correlation	Sig. (2-Tailed)	N	Pearson Correlation	Sig. (2-Tailed)	N
COPSOQ	Job satisfaction (scale)					
	0.40 **	0.000	970	0.54 **	0.000	971
Adapted from CBI	Patient-related burnout (scale)					
	−0.35 **	0.000	970	−0.30 **	0.000	971
TLI short	Transformational leadership (scale)					
	0.21 **	0.000	940	0.39 **	0.000	949
TWINS Occupational Safety versus TWINS Patient Safety	Supervisor support for occupational safety (scale)			Supervisor support for patient safety (scale)		
	0.23 **	0.000	940	0.57 **	0.000	949
	Occupational safety-related behaviour of the direct supervisor (factor)			Patient safety-related behaviour of the direct supervisor (factor)		
	0.20 **	0.000	940	0.45 **	0.000	949
	Occupational safety-related behaviour of the hospital management (factor)			Patient safety-related behaviour of the hospital management (factor)		
	0.24 **	0.000	940	0.55 **	0.000	949
	Individual influence on how well occupational safety is implemented at the workplace (single item)			Individual influence on how well patient safety is implemented at the workplace (single item)		
	0.21 **	0.000	940	0.46 **	0.000	949

^1^ Dependent variable: personal perception of the frequency of occupational risks (index). ^2^ Dependent variable: perceived patient safety (factor). ** Correlation is significant at the 0.01 level (two-tailed).

## Supplementary Materials

The following table is available online at http://www.mdpi.com/1660-4601/15/10/2131/s1, Table S1: Additional correlations of independent variables (COPSOQ, adapted CBI, TLI short, TWINS) with the two dependent variables.
