# Supplementary material for: Do Occupational and Patient Safety Culture in Hospitals Share Predictors in the Field of Psychosocial Working Conditions? Findings from a Cross-Sectional Study in German University Hospitals
Source: Int J Environ Res Public Health. 2018 Sep 27;15(10):2131. doi: 10.3390/ijerph15102131 (PMC6210136; doi:10.3390/ijerph15102131)
Supplement: Supplementary file 1 [file ijerph-15-02131-s001.pdf]

Table S1. Additional correlations of independent variables (COPSOQ, adapted CBI, TLI short, TWINS) with the two dependent variables.

**Independent variables (scales, factors and single items)**	**Dependent variable** [1]	
	Pearson correlation with the dependent variable[1] for Occupational Safety Culture	Pearson correlation with the dependent variable [2] for Patient Safety Culture
COPSOQ—Psychosocial working conditions		
Quantitative demands (scale)	–0.25 **	–0.30 **
Emotional demands (scale)	–0.23 **	–0.17 **
Work–privacy conflict (scale)	–0.33 **	–0.28 **
Influence at work (scale)	0.23 **	0.32 **
Degree of freedom at work (scale)	0.28 **	0.31 **
Possibilities for development (scale)	0.12 **	0.24 **
Meaning of work (scale)	0.22 **	0.31 **
Workplace commitment (scale)	0.19 **	0.34 **
Predictability (scale)	0.26 **	0.43 **
Role clarity (scale)	0.21 **	0.33 **
Role conflicts (scale)	–0.31 **	–0.42 **
Social relations (scale)	0.06 *	0.08 *
Feedback (scale)	0.09 **	0.26 **
Social support (scale)	0.17 **	0.29 **
Sense of community (scale)	0.17 **	0.23 **
Quality of leadership (scale)	0.23 **	0.41 **
Job satisfaction (scale)	0.40 **	0.54 **
Adapted from CBI—Psychosocial working conditions		
Patient-related burnout (scale)	–0.35 **	–0.30 **
TLI short—Transformational leadership		
Transformational leadership (scale)	0.21**	0.39 **
TWINS Occupational Safety—Patient Safety		
Supervisor support (scale)	0.23 **	0.57 **
Safety-related behaviour of the direct supervisor (factor)	0.20 **	0.45 **
Safety-related behaviour of the hospital management (factor)	0.24 **	0.55 **
Individual influence on implementation at the workplace (single item)	0.21 **	0.46 **

[1] Dependent variable: personal perception of the frequency of occupational risks (index)

[2] Dependent variable: perceived patient safety (factor)

* Correlation is significant at the 0.05 level (two-tailed)

** Correlation is significant at the 0.01 level (two-tailed)
